# Supplementary material for: Furin cleavage of the SARS-CoV-2 spike is modulated by O-glycosylation
Source: bioRxiv. 2021 Feb 5:2021.02.05.429982. Preprint. [Version 1] doi: 10.1101/2021.02.05.429982 (PMC7872346; doi:10.1101/2021.02.05.429982)
Supplement: Supplement 1 [file media-1.pdf]

Supplementary Figures for

Furin cleavage of the SARS-CoV-2 spike is modulated by O-glycosylation

Liping Zhang[1], Matthew Mann[2], Zulfeqhar Syed[1], Hayley Reynolds[1], E Tian[1], Nadine L. Samara[3], Darryl C. Zeldin[4], Lawrence A. Tabak[2], and Kelly G. Ten Hagen[1,*]

Correspondence to: Kelly.Tenhagen@nih.gov

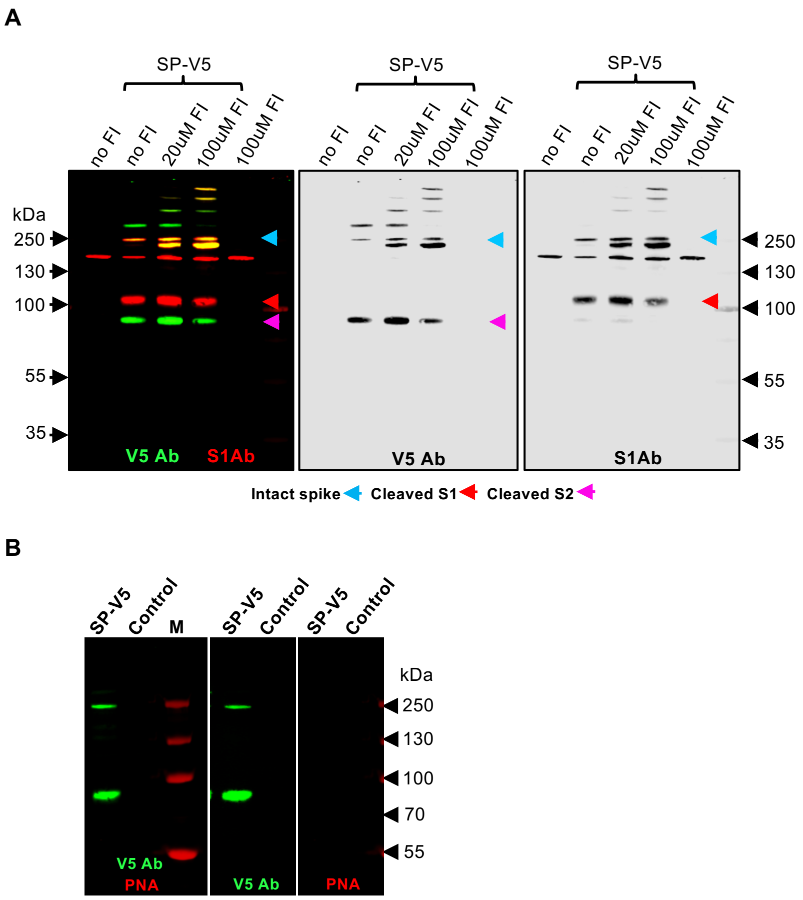

Fig. S1. Furin cleavage of S expressed in *Drosophila* S2R+ cells. (**A**) Decreased cleavage of S expressed in *Drosophila* S2R+ cells is seen upon incubation with increased concentrations of a furin inhibitor (FI). V5 Ab was used to detect intact S (blue arrow) and the cleaved S2 fragment (pink arrow). The S1 Ab was used to detect the cleaved S1 fragment (red arrow). (**B**). S expressed in *Drosophila* S2R+ cells (as detected by V5 Ab) is not PNA reactive (red). Size markers (kDa) are shown to the left and right of each blot.

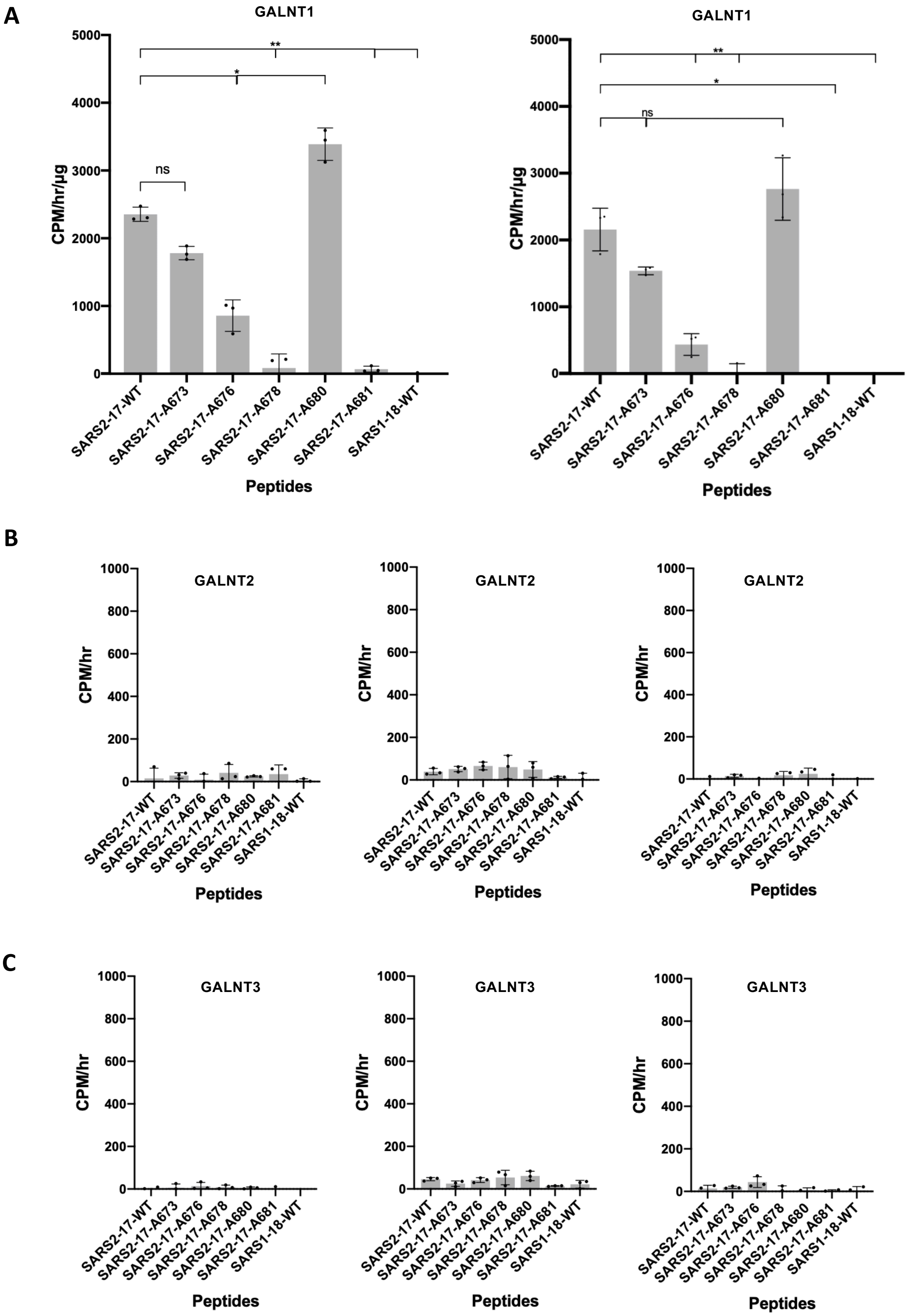
A
GALNT1
GALNT1
CPM/hr/µg
ns
*
**
SARS2-17-WT
SARS2-17-A673
SARS2-17-A676
SARS2-17-A678
SARS2-17-A680
SARS2-17-A681
SARS1-18-WT
Peptides
B
GALNT2
CPM/hr
C
GALNT3
CPM/hr

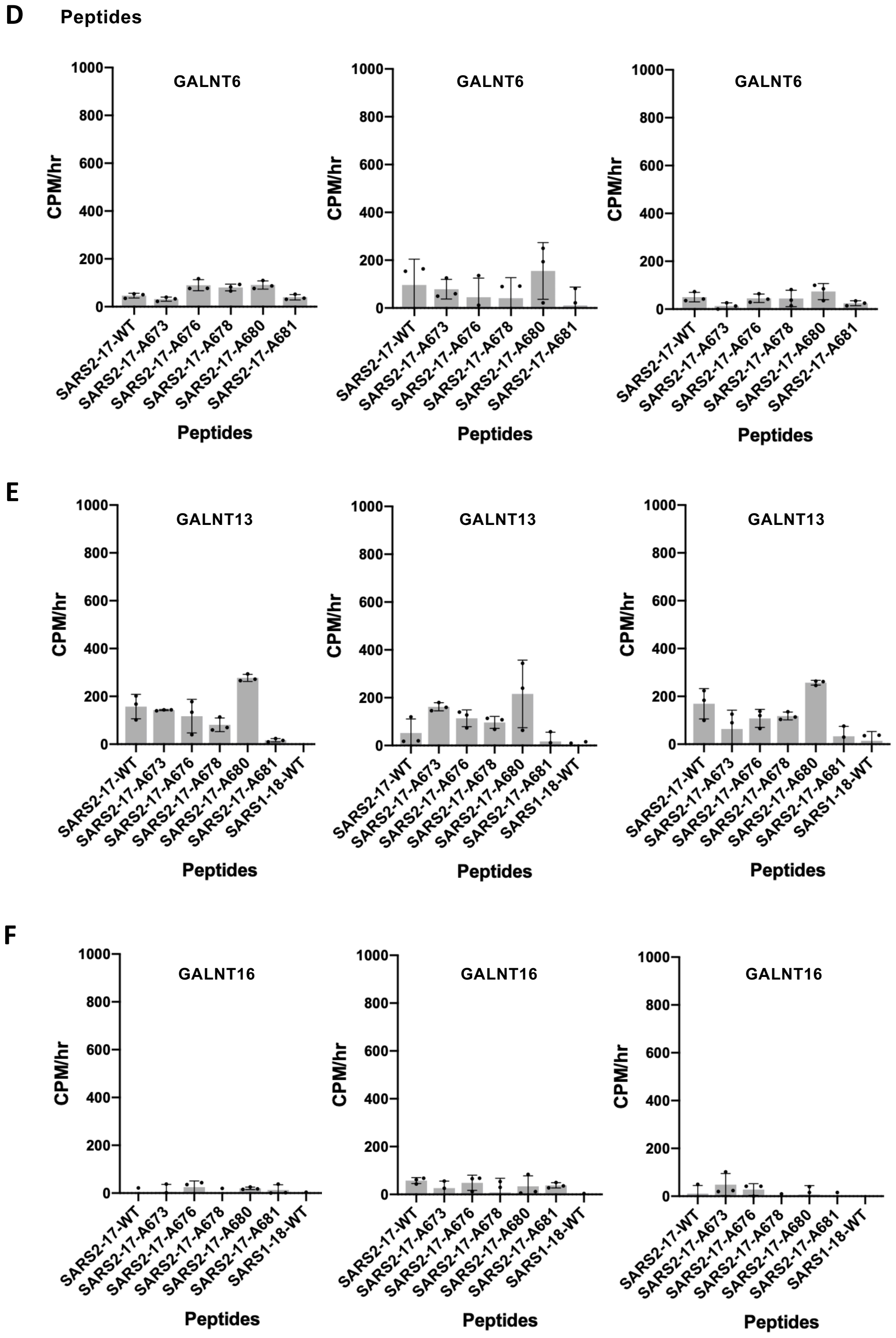

Fig. S2. O-glycosylation of SARS-CoV-2 S by GALNT1. Enzyme assays testing for O-glycosylation of peptides and mutated variants from the furin proximal region of the SARS-CoV-2 S and the related region of SARS-CoV-1 S using GALNT1 (**A**), GALNT2 (**B**), GALNT3

(**C**), GALNT6 (**D**), GALNT13 (**E**), and GALNT16 (**F**). GALNT1 activity is dependent on the unique proline at position 681 (P681). Peptide sequences are as shown in the main text. Each data point represents an individual assay. Error bars are SD.*$P<0.05$;**$P<0.01$.

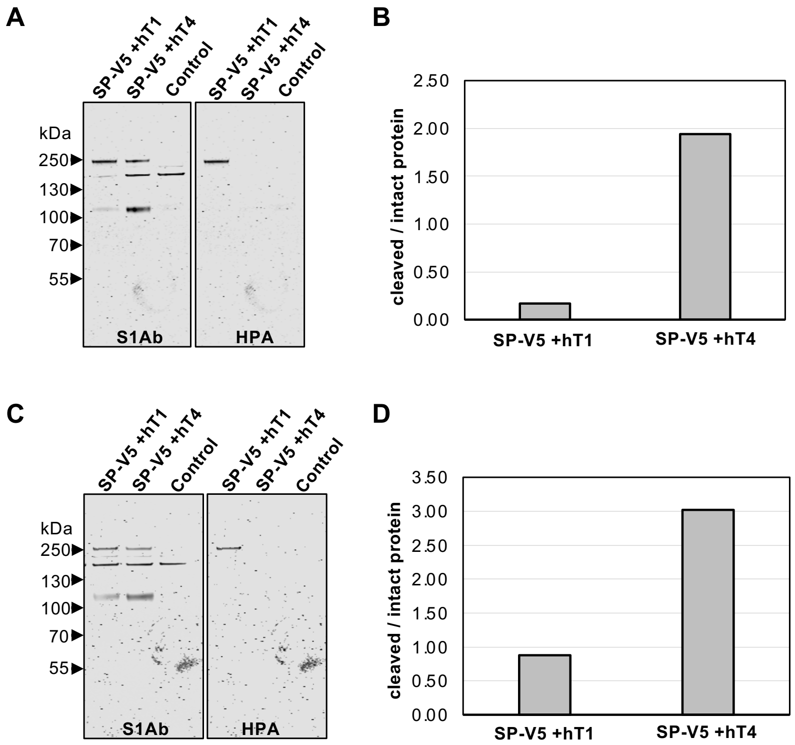

Fig. S3. O-glycosylation of SARS-CoV-2 S decreases furin cleavage in *Drosophila* S2R+ cells. Repeats of experiments coexpressing S with GALNT1 (hT1) (which glycosylates S) or GALNT4 (hT4) (which does not glycosylate S) in *Drosophila* S2R+ cells. S1Ab was used to detect cleaved and uncleaved S via westerns (**A** and **C**). O-glycosylation is seen (HPA staining) only on the intact S coexpressed with GALNT1. Ratios of cleaved to uncleaved S were quantitated (**B** and **D**).

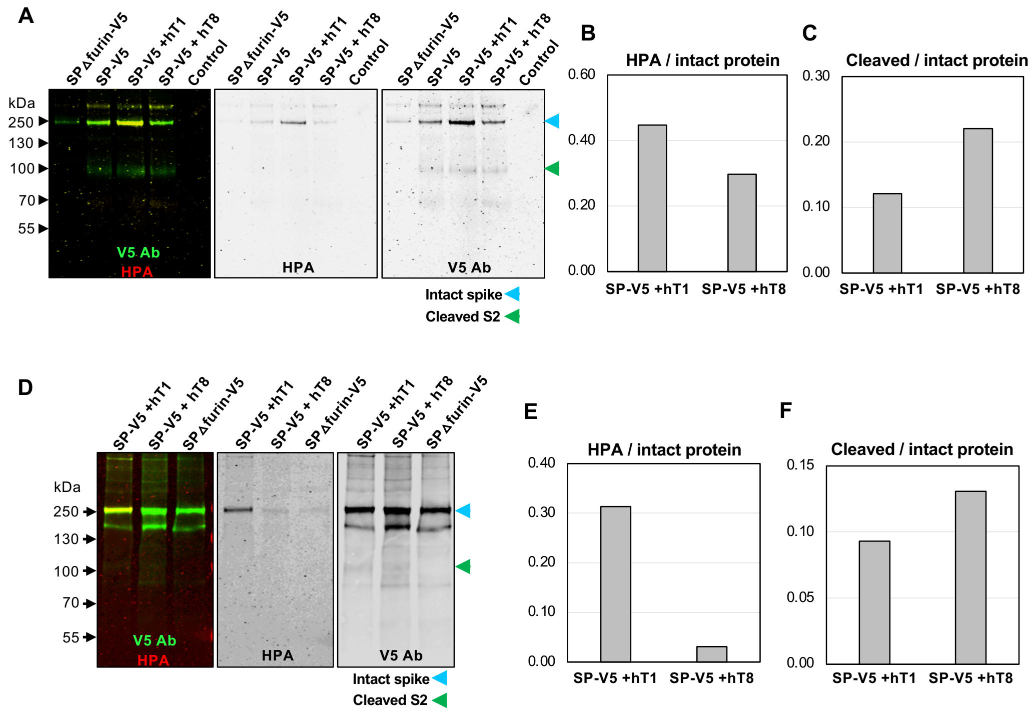

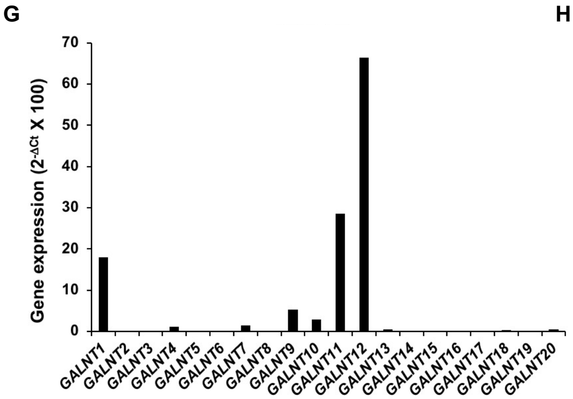

H

Gene	CT	SD
hS29	19.14	0.12
GALNT1	24.94	0.07
GALNT2	ND	NA
GALNT3	33.64	0.52
GALNT4	29.02	0.20
GALNT5	36.36	0.62
GALNT6	36.22	1.09
GALNT7	28.58	0.07
GALNT8	ND	NA
GALNT9	26.69	0.43
GALNT10	27.60	0.08
GALNT11	24.27	0.03
GALNT12	23.05	0.04
GALNT13	30.34	0.44
GALNT14	ND	NA
GALNT15	ND	NA
GALNT16	33.35	0.70
GALNT17	34.12	NA
GALNT18	30.80	0.16
GALNT19	37.65	0.78
GALNT20	30.28	0.30

Fig. S4. O-glycosylation of SARS-CoV-2 S decreases furin cleavage in Vero E6 cells. Repeats of experiments coexpressing S with GALNT1 (hT1) (which glycosylates S) or GALNT8 (hT8) (which does not glycosylate S) Vero E6 cells. V5 Ab was used to detect cleaved and uncleaved S via westerns (**A** and **D**). O-glycosylation (HPA staining) is increased on intact S coexpressed with GALNT1 relative to GALNT8 (**B** and **E**). Ratios of cleaved to uncleaved S from each experiment were quantitated (**C** and **F**). Endogenous expression of *GALNT* family members in Vero E6 cells was assessed by QPCR (**G**). Cycle threshold (CT) values (**F**) are shown. Error bars are SD. ND, not detected. NA, not available.

Table S1.
PCR primer sequences

Gene	Sense primer sequence	Anti-sense primer sequence
GALNT1	GCAGGCAGTGCTATTTCTTGTG	CTGCTTCCTTTCAAATTTCCTGGG
GALNT2	CCTCTCAGCCTTCCTATCATCC	CGCAACCGCAGTAAGTCACT
GALNT3	CTCGGTTCTGCCTCTCCA	ACAGTTGCGGCTCAGTAGA
GALNT4	GCGGTGAGGTGGACTTGG	GGAGGTCTGAGAGCCTTCTTGA
GALNT5	CCACGCAGGCAGAGACTGACAA	CAGCAGCAGCAGCAGCAGAG
GALNT6	GGGAGGGTTCAGGGCAGCAATG	GGGAGGGTGGGCGTAGAGATGG
GALNT7	GGCTAGTGGTCCTCTGGTCTTCC	CTCTGTCTTCCCTCATCCTGCTCA
GALNT8	CTCCAGACCTGTAGCACGCAAGT	TGATGACCCAGATCCAGCACCAT
GALNT9	GGCAGACACAGCAGGACA	TGTTTGAGTTGGCATCACTGTC
GALNT10	AAGGAGCCAGCCAGGTGTAATAC	CAGTTGTGATGATGGTGGGAGGTT
GALNT11	GCCACGGGTCAGGAGGAT	TGAAGAGGAGCCATCGCAGAT
GALNT12	CAGGCTGCGAGGAAGGAGTC	GGTGGGTTCTGGTCACTGCTTAG
GALNT13	CGTCCACACCACTCACCACACAT	TGGGGCAGAGGGAACAAAACACT
GALNT14	TCGTCGTCAACCCATGTGAGTCCT	GCTGCCCAGTTTCCAGTCTGTTCT
GALNT15	AACACTGGACTTGGGCTCTG	CACCTGCTCCTGCCTGAC
GALNT16	CCTGAACCTCTGCTCTGGATTG	TGCGGACCACAGACACTTG
GALNT17	AACAGCGGAGCGTGAGAG	GGAGTCCAGCCAGGAAGTG
GALNT18	CACCGTGGATGATGATGACAAC	AACTCCAGGTCGCTATTCTCC
GALNT19	AGACGCCTTCCACGAGAT	CACGATGTCCTCCAGCAG
GALNT20	CCTCCTGTGTCTCATCTCTTGC	TGGTCCTCACTGCCTCCTT
